# Supplementary material for: A yeast phenomic model for the influence of Warburg metabolism on genetic buffering of doxorubicin
Source: Cancer Metab. 2019 Oct 23;7:9. doi: 10.1186/s40170-019-0201-3 (PMC6806529; doi:10.1186/s40170-019-0201-3)

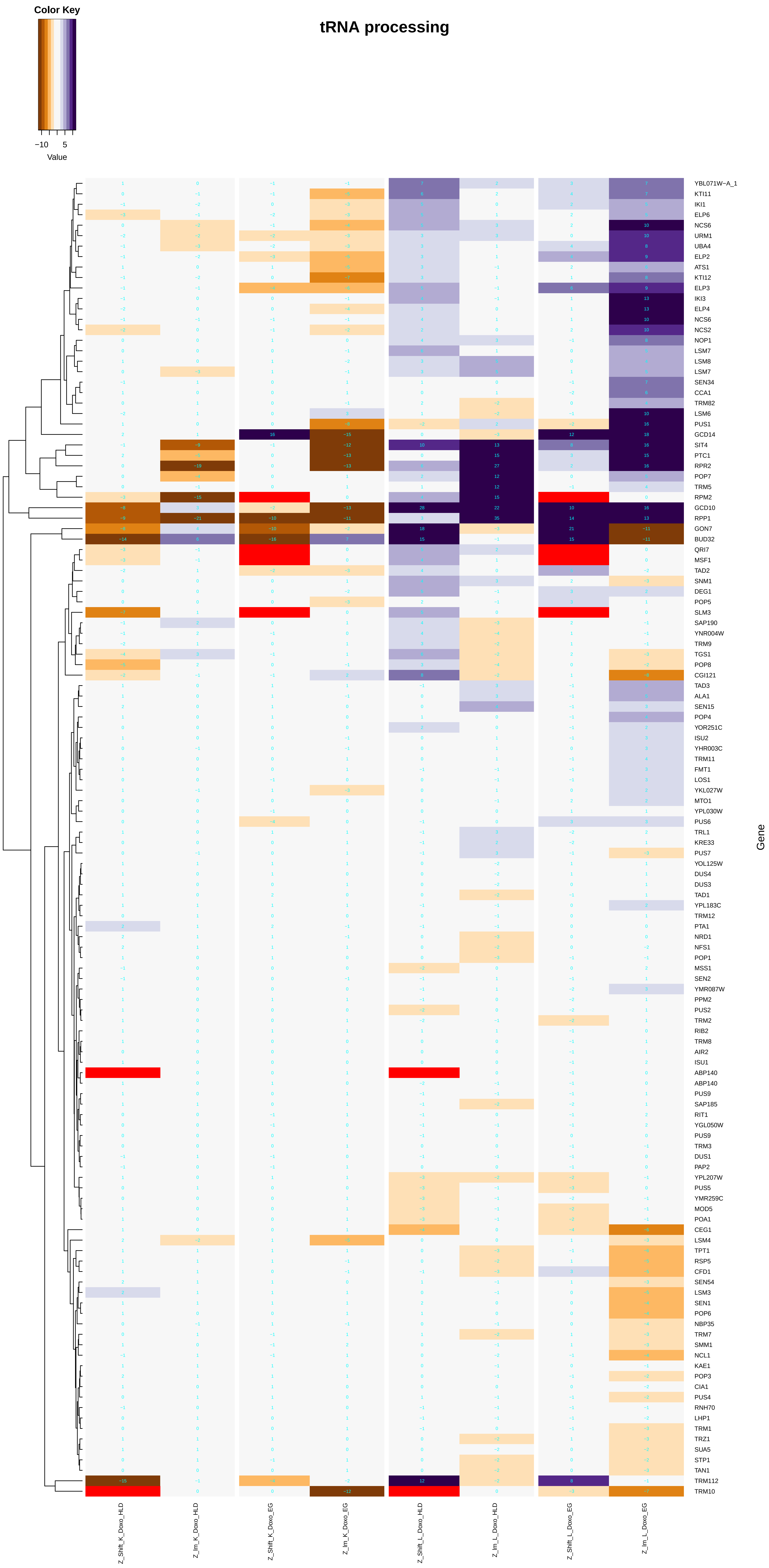
Color Key
−10
5
Value
tRNA processing
YBL071W−A_1
KTI11
IKI1
ELP6
NCS6
URM1
UBA4
ELP2
ATS1
KTI12
ELP3
IKI3
ELP4
NCS6
NCS2
NOP1
LSM7
LSM8
LSM7
SEN34
CCA1
TRM82
LSM6
PUS1
GCD14
SIT4
PTC1
RPR2
POP7
TRM5
RPM2
GCD10
RPP1
GON7
BUD32
QRI7
MSF1
TAD2
SNM1
DEG1
POP5
SLM3
SAP190
YNR004W
TRM9
TGS1
POP8
CGI121
TAD3
ALA1
SEN15
POP4
YOR251C
ISU2
YHR003C
TRM11
FMT1
LOS1
YKL027W
MTO1
YPL030W
PUS6
TRL1
KRE33
PUS7
YOL125W
DUS4
DUS3
TAD1
YPL183C
TRM12
PTA1
NRD1
NFS1
POP1
MSS1
SEN2
YMR087W
PPM2
PUS2
TRM2
RIB2
TRM8
AIR2
ISU1
ABP140
ABP140
PUS9
SAP185
RIT1
YGL050W
PUS9
TRM3
DUS1
PAP2
YPL207W
PUS5
YMR259C
MOD5
POA1
CEG1
LSM4
TPT1
RSP5
CFD1
SEN54
LSM3
SEN1
POP6
NBP35
TRM7
SMM1
NCL1
KAE1
POP3
CIA1
PUS4
RNH70
LHP1
TRM1
TRZ1
SUA5
STP1
TAN1
TRM112
TRM10
Gene
Z_Shift_K_Doxo_HLD
Z_lm_K_Doxo_HLD
Z_Shift_K_Doxo_EG
Z_lm_K_Doxo_EG
Z_Shift_L_Doxo_HLD
Z_lm_L_Doxo_HLD
Z_Shift_L_Doxo_EG
Z_lm_L_Doxo_EG

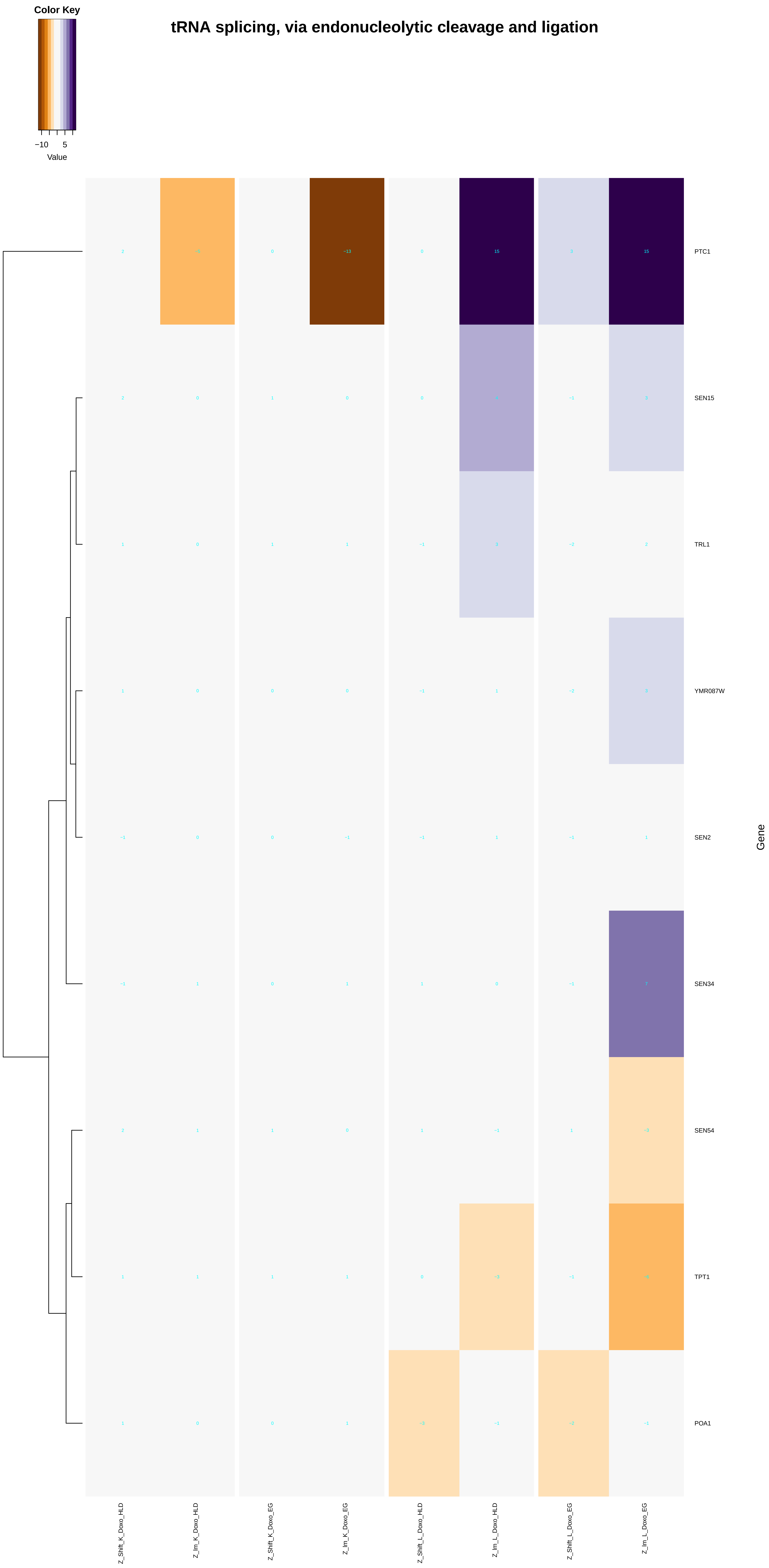
tRNA splicing, via endonucleolytic cleavage and ligation
Color Key
−10
5
Value
PTC1
SEN15
TRL1
YMR087W
SEN2
SEN34
SEN54
TPT1
POA1
Gene
Z_Shift_K_Doxo_HLD
Z_lm_K_Doxo_HLD
Z_Shift_K_Doxo_EG
Z_lm_K_Doxo_EG
Z_Shift_L_Doxo_HLD
Z_lm_L_Doxo_HLD
Z_Shift_L_Doxo_EG
Z_lm_L_Doxo_EG

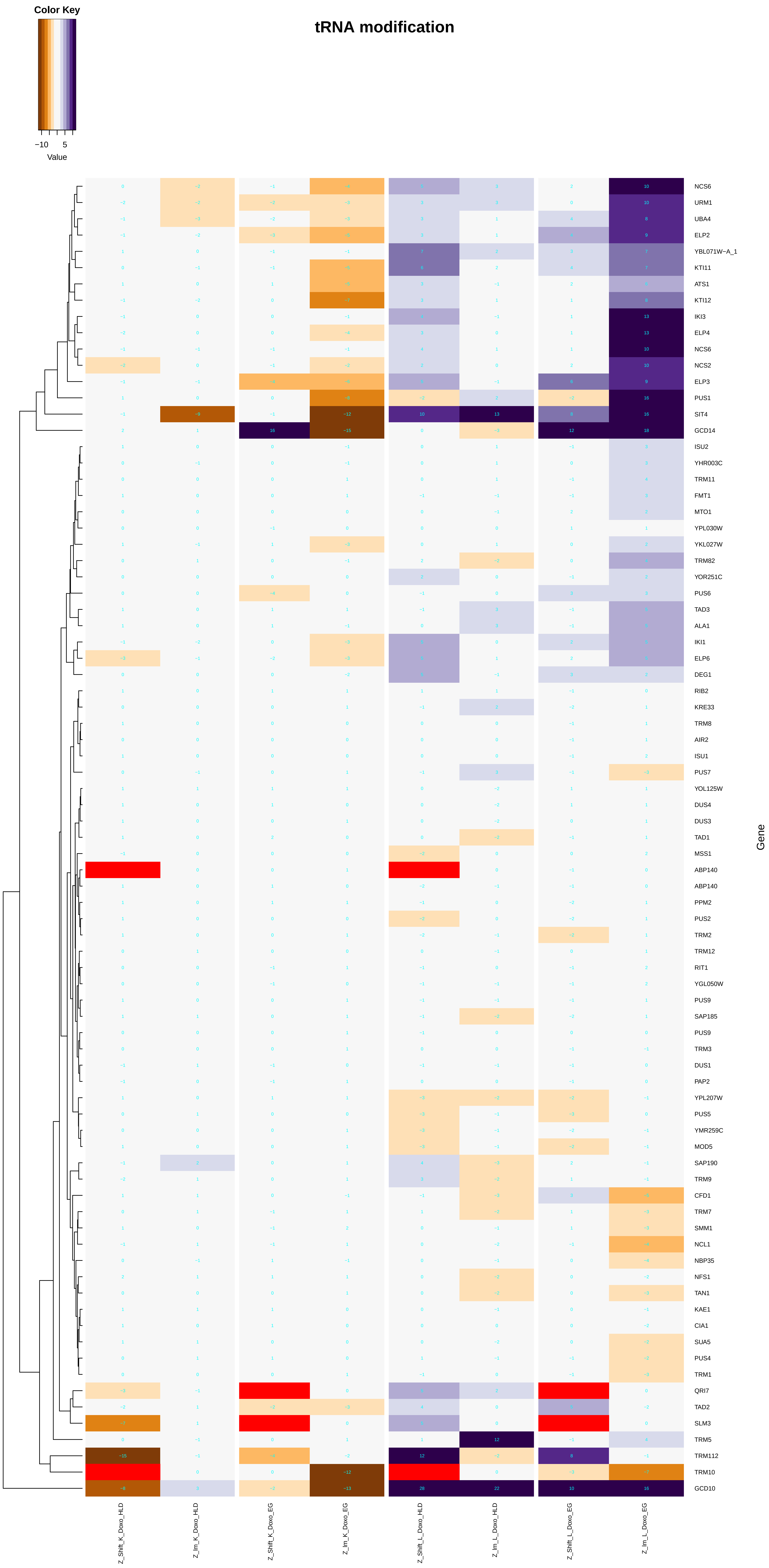

tRNA modification
Color Key
−10 5
Value
Gene
NCS6
URM1
UBA4
ELP2
YBL071W−A_1
KTI11
ATS1
KTI12
IKI3
ELP4
NCS6
NCS2
ELP3
PUS1
SIT4
GCD14
ISU2
YHR003C
TRM11
FMT1
MTO1
YPL030W
YKL027W
TRM82
YOR251C
PUS6
TAD3
ALA1
IKI1
ELP6
DEG1
RIB2
KRE33
TRM8
AIR2
ISU1
PUS7
YOL125W
DUS4
DUS3
TAD1
MSS1
ABP140
ABP140
PPM2
PUS2
TRM2
TRM12
RIT1
YGL050W
PUS9
SAP185
PUS9
TRM3
DUS1
PAP2
YPL207W
PUS5
YMR259C
MOD5
SAP190
TRM9
CFD1
TRM7
SMM1
NCL1
NBP35
NFS1
TAN1
KAE1
CIA1
SUA5
PUS4
TRM1
QRI7
TAD2
SLM3
TRM5
TRM112
TRM10
GCD10
Z_Shift_K_Doxo_HLD
Z_lm_K_Doxo_HLD
Z_Shift_K_Doxo_EG
Z_lm_K_Doxo_EG
Z_Shift_L_Doxo_HLD
Z_lm_L_Doxo_HLD
Z_Shift_L_Doxo_EG
Z_lm_L_Doxo_EG

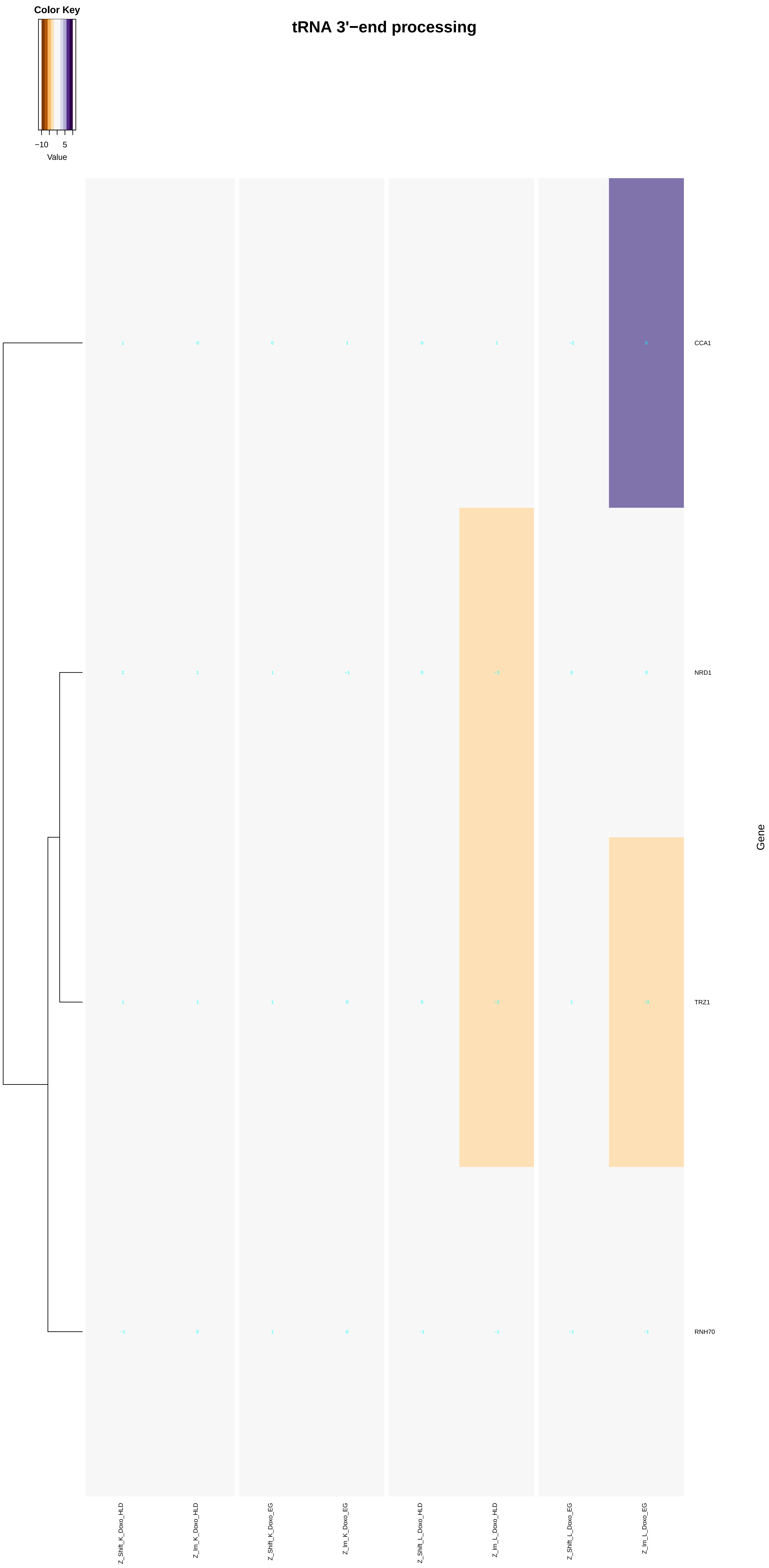
tRNA 3'−end processing
Color Key
−10
5
Value
CCA1
NRD1
TRZ1
RNH70
Gene
Z_Shift_K_Doxo_HLD
Z_Im_K_Doxo_HLD
Z_Shift_K_Doxo_EG
Z_Im_K_Doxo_EG
Z_Shift_L_Doxo_HLD
Z_Im_L_Doxo_HLD
Z_Shift_L_Doxo_EG
Z_Im_L_Doxo_EG

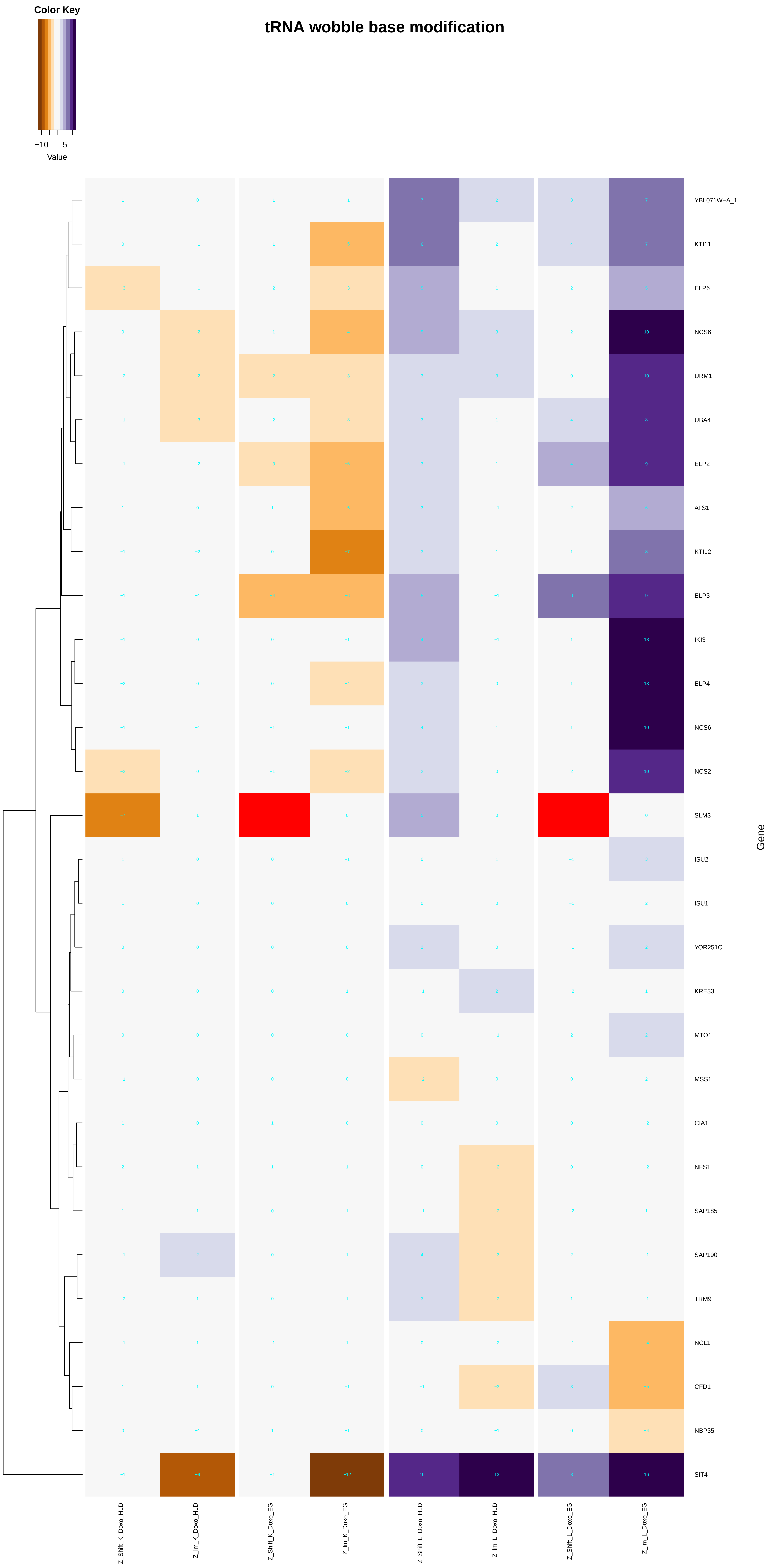

tRNA wobble base modification
Color Key
−10
5
Value
YBL071W−A_1
KTI11
ELP6
NCS6
URM1
UBA4
ELP2
ATS1
KTI12
ELP3
IKI3
ELP4
NCS6
NCS2
SLM3
ISU2
ISU1
YOR251C
KRE33
MTO1
MSS1
CIA1
NFS1
SAP185
SAP190
TRM9
NCL1
CFD1
NBP35
SIT4
Gene
Z_Shift_K_Doxo_HLD
Z_Im_K_Doxo_HLD
Z_Shift_K_Doxo_EG
Z_Im_K_Doxo_EG
Z_Shift_L_Doxo_HLD
Z_Im_L_Doxo_HLD
Z_Shift_L_Doxo_EG
Z_Im_L_Doxo_EG

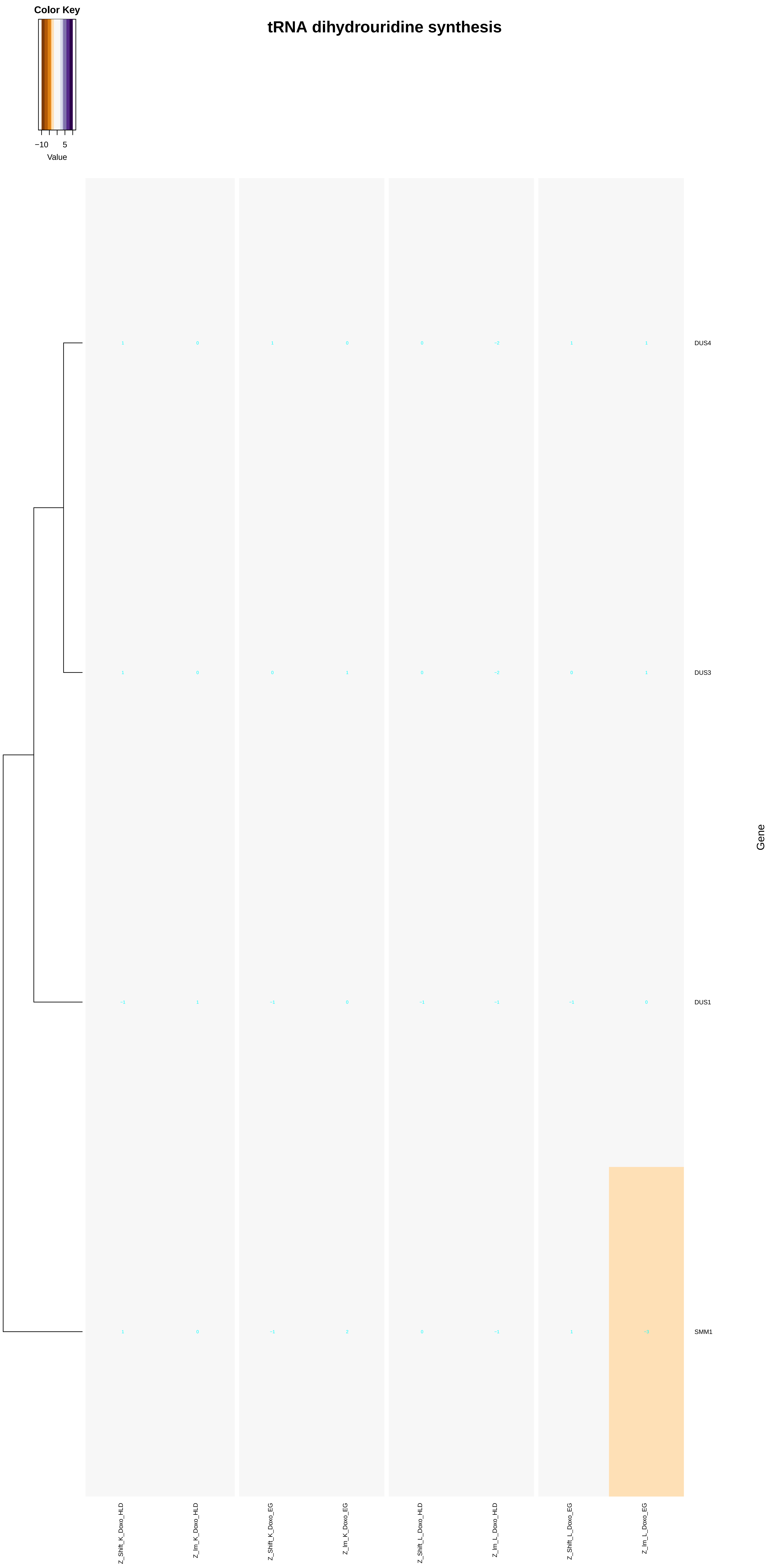

Color Key
−10
5
Value
tRNA dihydrouridine synthesis
DUS4
DUS3
DUS1
SMM1
Gene
Z_Shift_K_Doxo_HLD
Z_Im_K_Doxo_HLD
Z_Shift_K_Doxo_EG
Z_Im_K_Doxo_EG
Z_Shift_L_Doxo_HLD
Z_Im_L_Doxo_HLD
Z_Shift_L_Doxo_EG
Z_Im_L_Doxo_EG

tRNA threonylcarbamoyladenosine modification

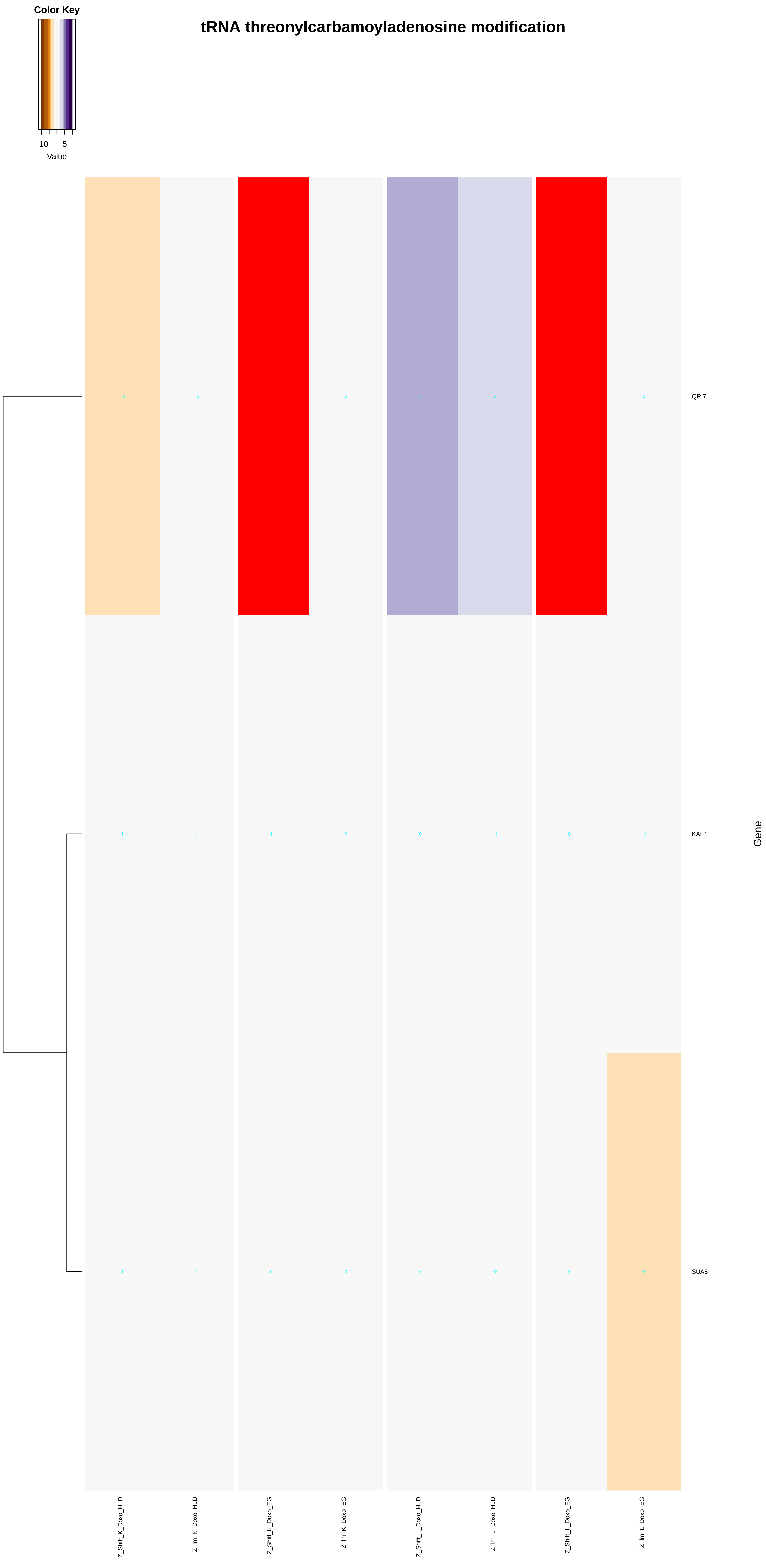

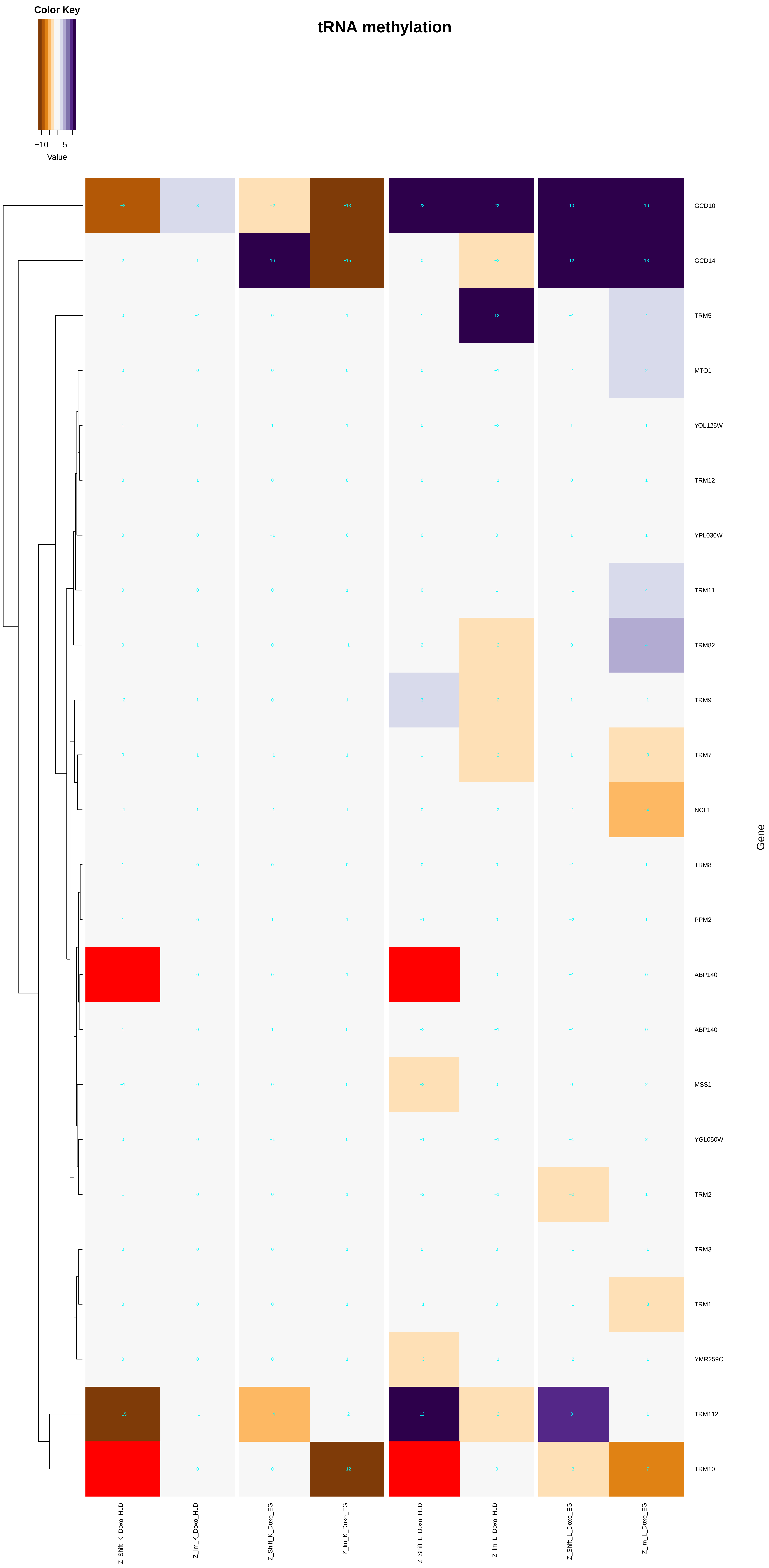

tRNA methylation
Color Key
−10
5
Value
GCD10
GCD14
TRM5
MTO1
YOL125W
TRM12
YPL030W
TRM11
TRM82
TRM9
TRM7
NCL1
TRM8
PPM2
ABP140
ABP140
MSS1
YGL050W
TRM2
TRM3
TRM1
YMR259C
TRM112
TRM10
Gene
Z_Shift_K_Doxo_HLD
Z_Im_K_Doxo_HLD
Z_Shift_K_Doxo_EG
Z_Im_K_Doxo_EG
Z_Shift_L_Doxo_HLD
Z_Im_L_Doxo_HLD
Z_Shift_L_Doxo_EG
Z_Im_L_Doxo_EG

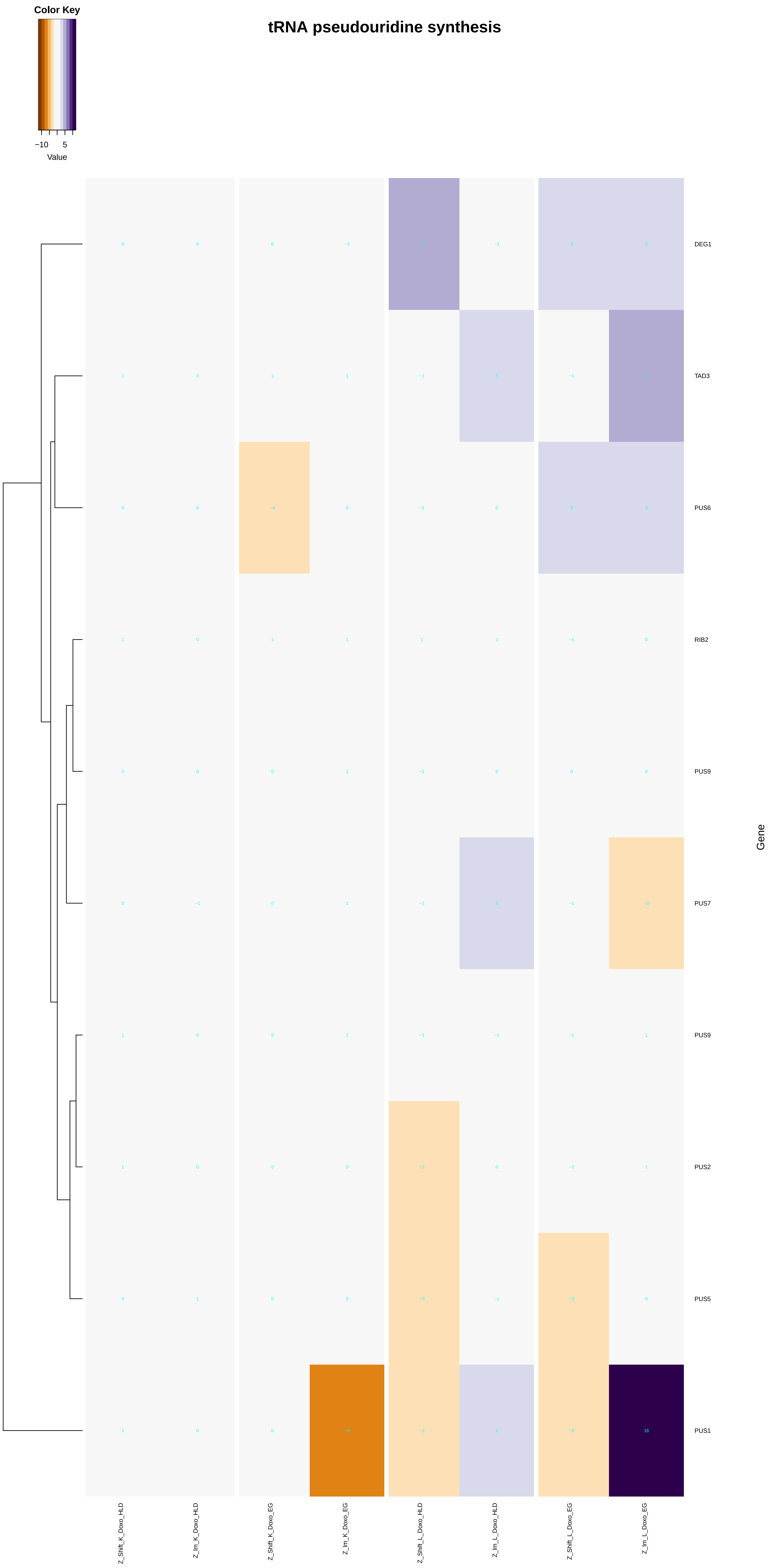

Color Key
−10
5
Value
tRNA pseudouridine synthesis
DEG1
TAD3
PUS6
RIB2
PUS9
PUS7
PUS9
PUS2
PUS5
PUS1
Gene
Z_Shift_K_Doxo_HLD
Z_Im_K_Doxo_HLD
Z_Shift_K_Doxo_EG
Z_Im_K_Doxo_EG
Z_Shift_L_Doxo_HLD
Z_Im_L_Doxo_HLD
Z_Shift_L_Doxo_EG
Z_Im_L_Doxo_EG

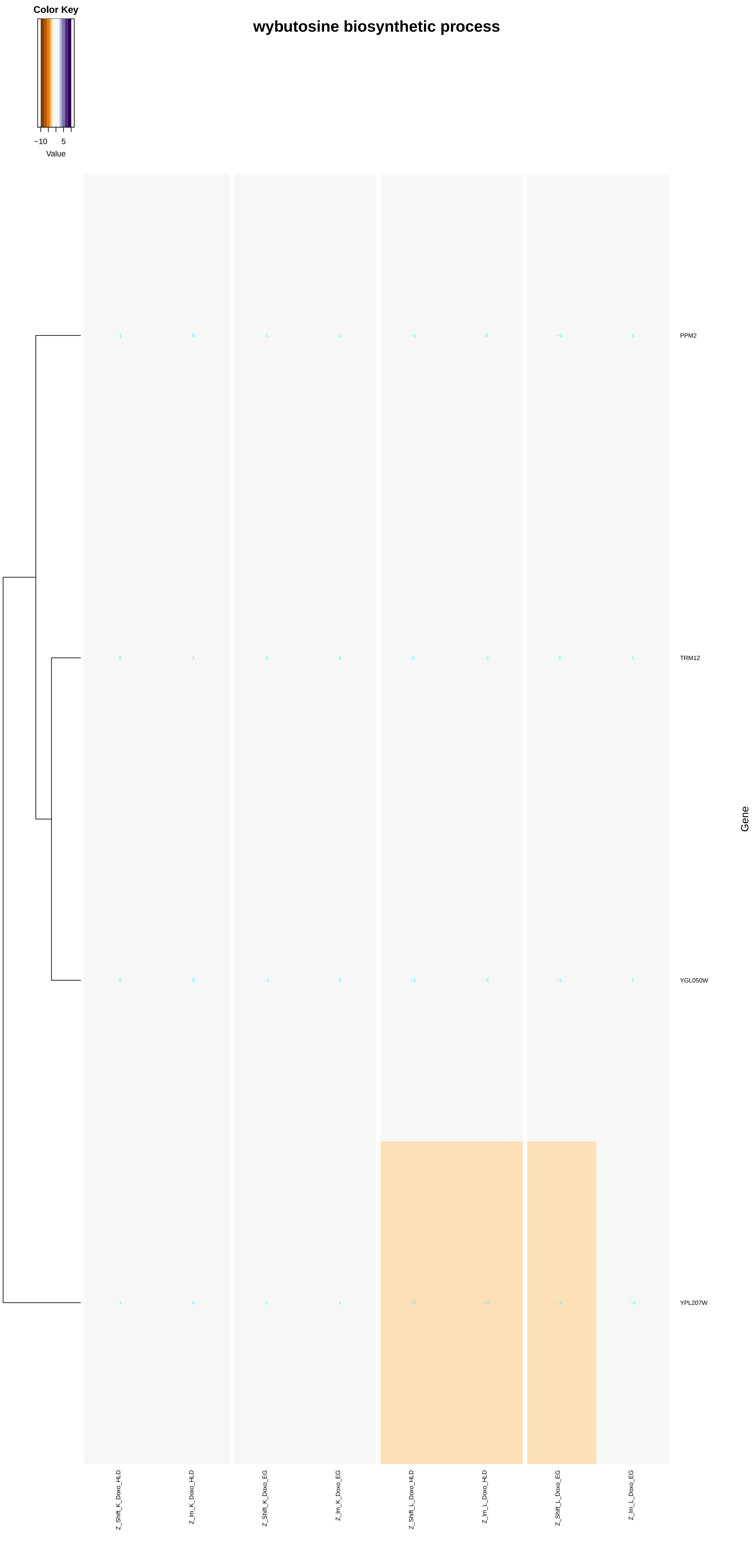
Color Key
wybutosine biosynthetic process
−10
5
Value
PPM2
TRM12
YGL050W
YPL207W
Gene
Z_Shift_K_Doxo_HLD
Z_lm_K_Doxo_HLD
Z_Shift_K_Doxo_EG
Z_lm_K_Doxo_EG
Z_Shift_L_Doxo_HLD
Z_lm_L_Doxo_HLD
Z_Shift_L_Doxo_EG
Z_lm_L_Doxo_EG

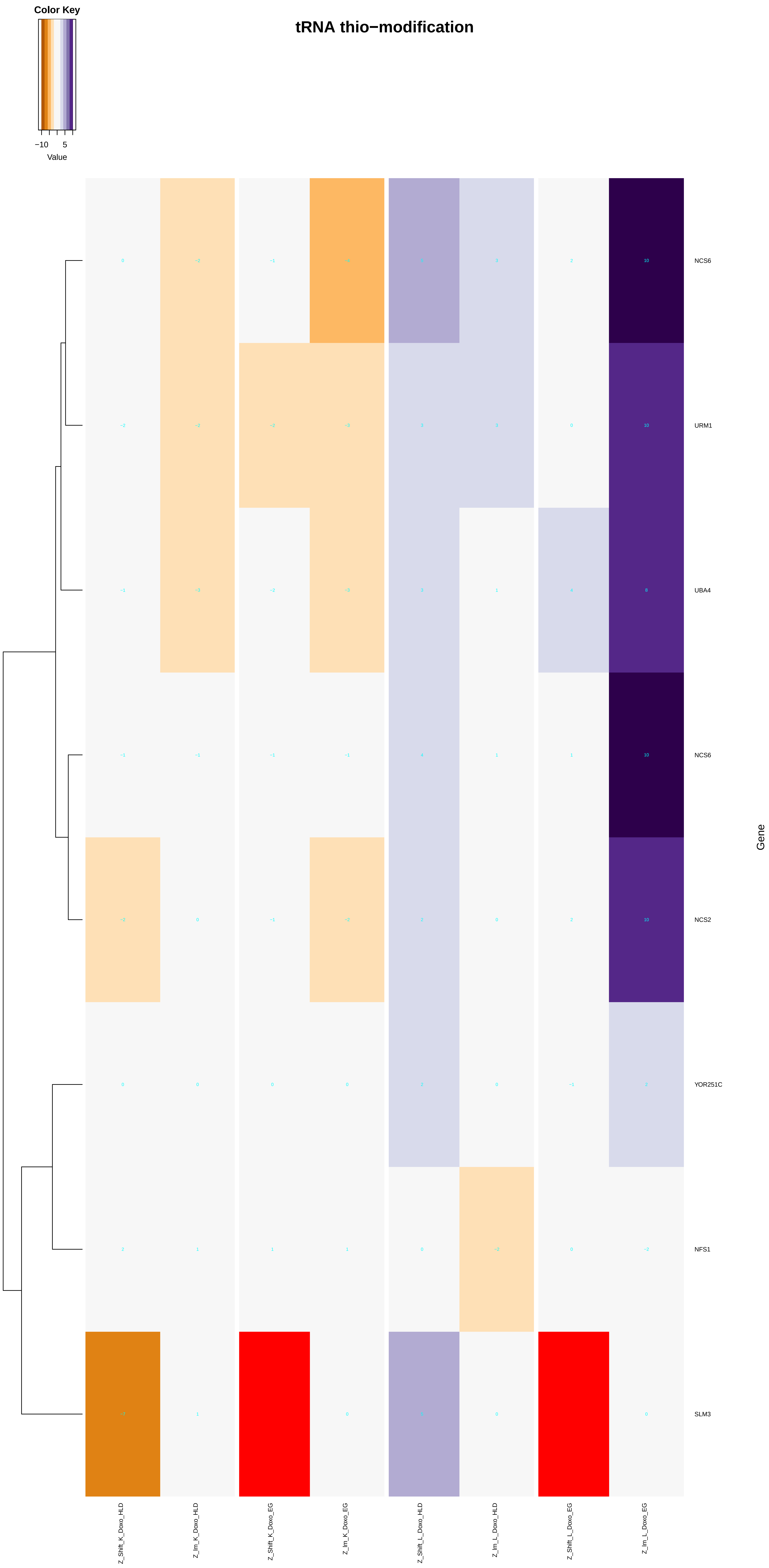
tRNA thio−modification
Color Key
−10
5
Value
NCS6
URM1
UBA4
NCS6
NCS2
YOR251C
NFS1
SLM3
Gene
Z_Shift_K_Doxo_HLD
Z_Im_K_Doxo_HLD
Z_Shift_K_Doxo_EG
Z_Im_K_Doxo_EG
Z_Shift_L_Doxo_HLD
Z_Im_L_Doxo_HLD
Z_Shift_L_Doxo_EG
Z_Im_L_Doxo_EG

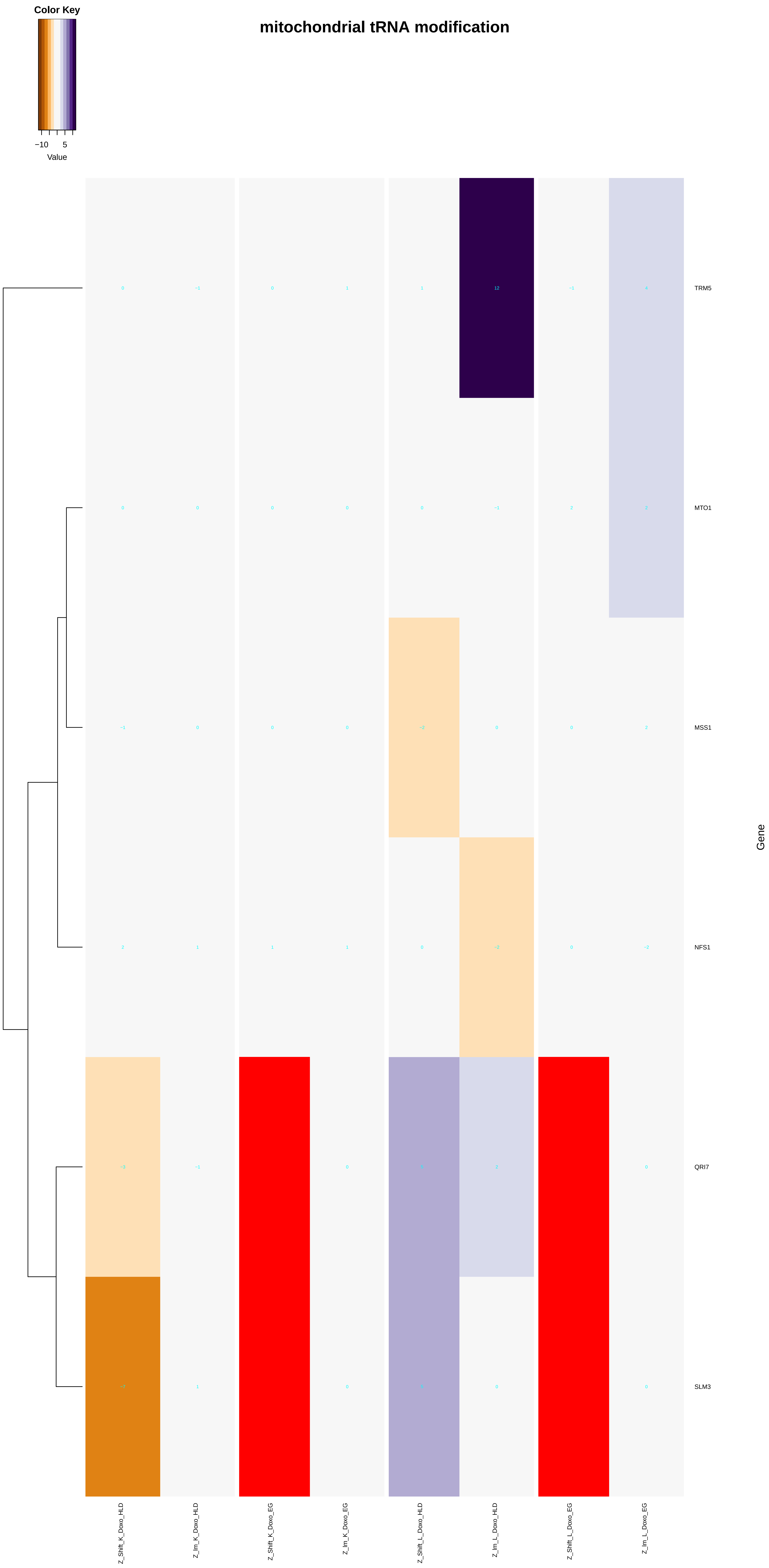
Color Key
−10
5
Value
mitochondrial tRNA modification
TRM5
MTO1
MSS1
NFS1
QRI7
SLM3
Gene
Z_Shift_K_Doxo_HLD
Z_lm_K_Doxo_HLD
Z_Shift_K_Doxo_EG
Z_lm_K_Doxo_EG
Z_Shift_L_Doxo_HLD
Z_lm_L_Doxo_HLD
Z_Shift_L_Doxo_EG
Z_lm_L_Doxo_EG

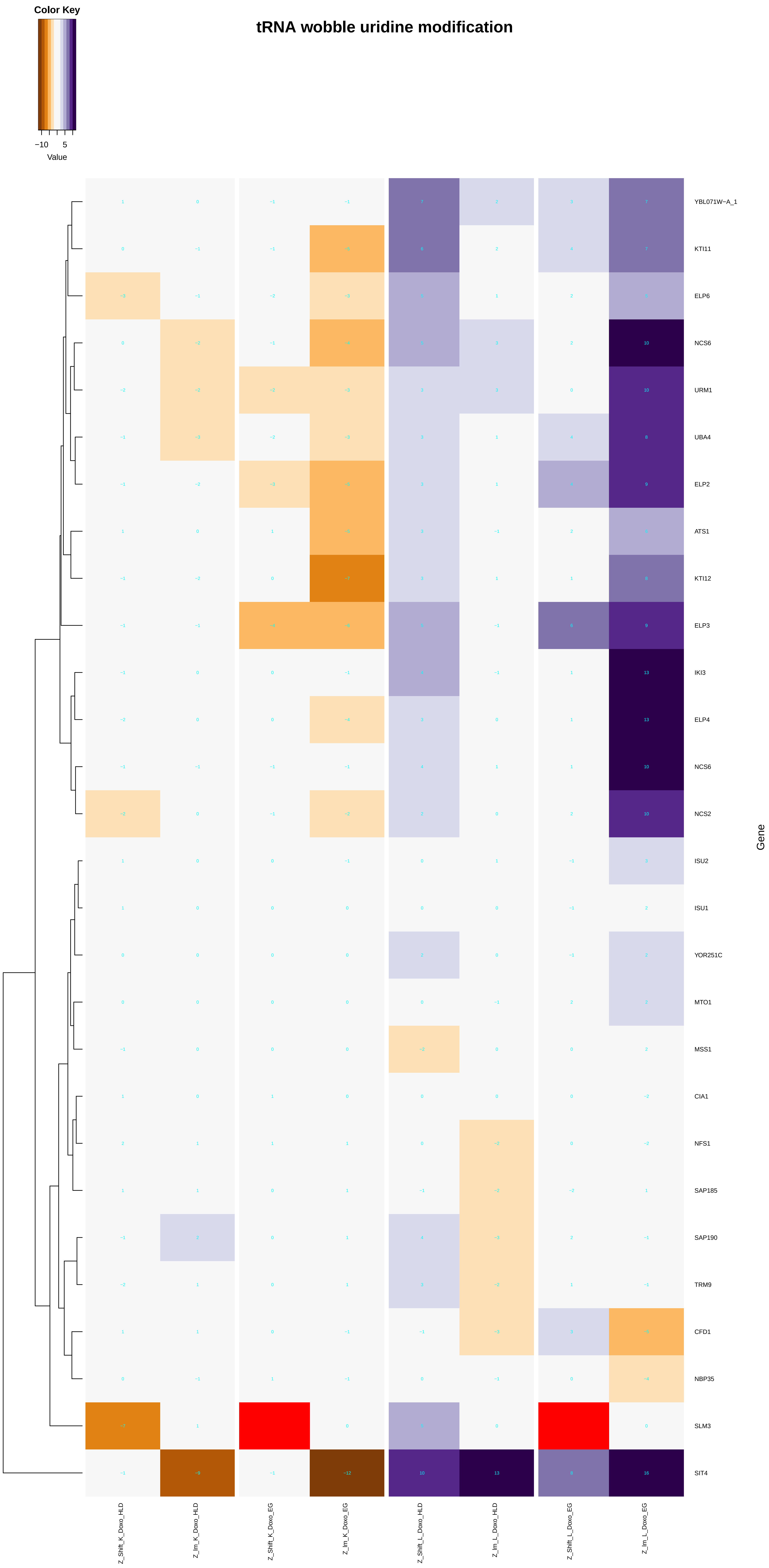

Color Key
−10
5
Value
tRNA wobble uridine modification
YBL071W−A_1
KTI11
ELP6
NCS6
URM1
UBA4
ELP2
ATS1
KTI12
ELP3
IKI3
ELP4
NCS6
NCS2
ISU2
ISU1
YOR251C
MTO1
MSS1
CIA1
NFS1
SAP185
SAP190
TRM9
CFD1
NBP35
SLM3
SIT4
Gene
Z_Shift_K_Doxo_HLD
Z_lm_K_Doxo_HLD
Z_Shift_K_Doxo_EG
Z_lm_K_Doxo_EG
Z_Shift_L_Doxo_HLD
Z_lm_L_Doxo_HLD
Z_Shift_L_Doxo_EG
Z_lm_L_Doxo_EG

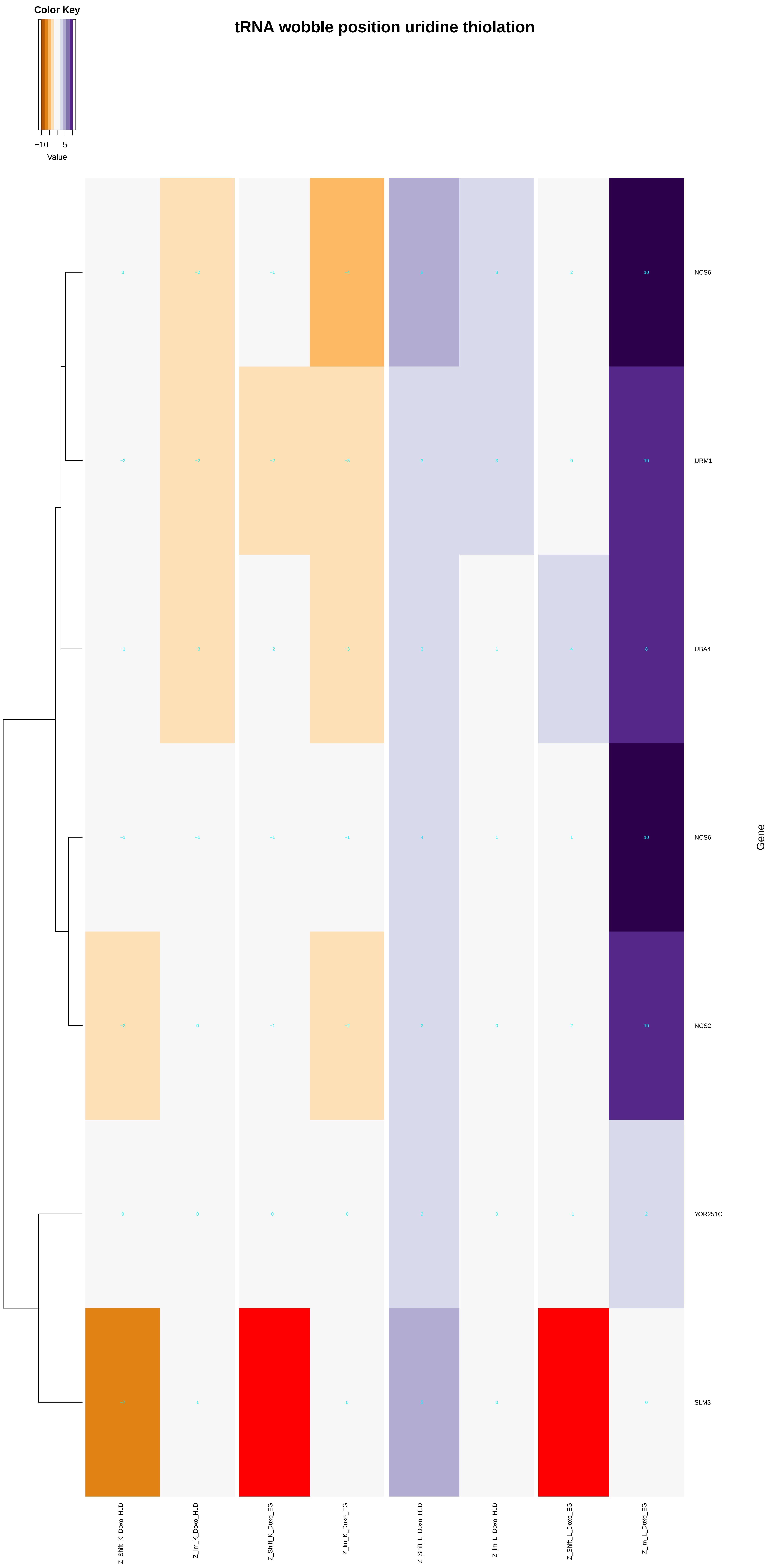

Color Key
−10
5
Value
tRNA wobble position uridine thiolation
NCS6
URM1
UBA4
NCS6
NCS2
YOR251C
SLM3
Gene
Z_Shift_K_Doxo_HLD
Z_lm_K_Doxo_HLD
Z_Shift_K_Doxo_EG
Z_lm_K_Doxo_EG
Z_Shift_L_Doxo_HLD
Z_lm_L_Doxo_HLD
Z_Shift_L_Doxo_EG
Z_lm_L_Doxo_EG

mitochondrial tRNA wobble uridine modification

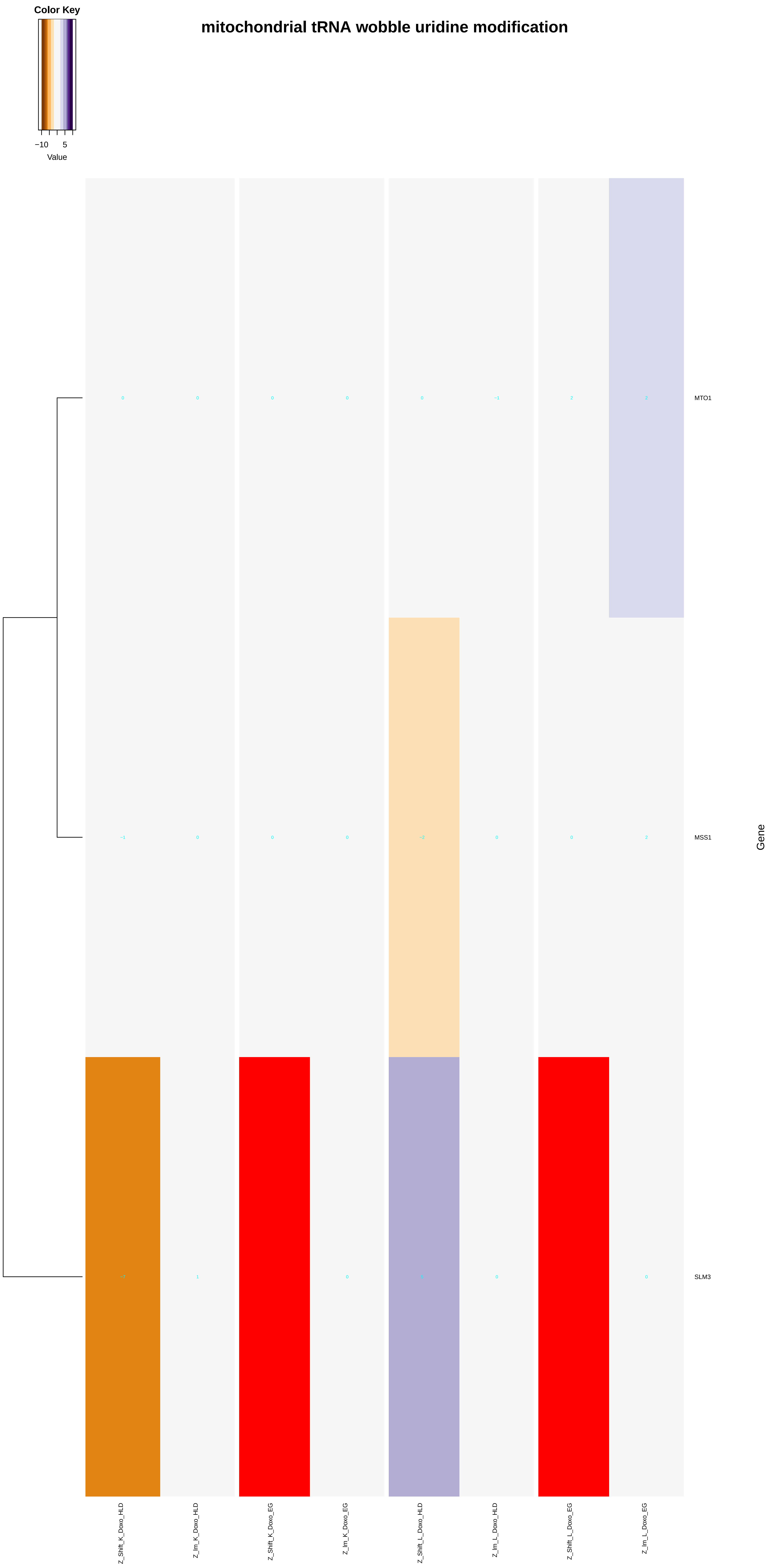

Supplement: Supplementary file 9 — Additional file 9. GO term-specific heatmaps for REMc/GTF-enriched clusters. GO term-specific heatmaps for significant GO process terms were generated as described in methods and Figs. 3 and 4. Any related child terms are presented in subsequent pages of the parent file name. GO terms with more than 100 children, with 2 or fewer genes annotated to the term, or a file size over 300KB are not shown. All heatmaps are generated with the same layout (see Figs. 3 and 4). [file 40170_2019_201_MOESM9_ESM.bz2 › Additional_File9_GOTermHeatmaps/Additional_File9_GOTermHeatmap/tRNA_processing.pdf]
